# Research on the spillover risk of antibiotic resistance gene from the gut microbiome of wild animal: A case study of the golden snub-nosed monkey

**DOI:** 10.1016/j.isci.2026.117624

**Published:** 2026-09-24

**Authors:** Sumei Liang, Jiaying Ma, Han Zhang, Hong Li, Shuzhen Zou, Dayong Li

**Affiliations:** 1 Key Laboratory of Southwest China Wildlife Resources Conservation of Ministry of Education, China West Normal University, Nanchong, China; 2 Key Laboratory of Conservation Biology of *Rhinopithecus Roxellana* at China West Normal University of Sichuan Province, China West Normal University, Nanchong, China

**Keywords:** antibiotic resistance genes, wild animals, collinearity analysis, human disturbances, spillover risk

## Abstract

Wild animals are “reservoirs” of antibiotic resistance genes (ARGs), and the interface of rivers between wild animals and humans is an ideal environment for spreading ARGs. In order to explore the risk of ARGs from wild animals to the human ecosystem through rivers, metagenomic sequencing was used to obtain the microbiome in the gut of golden snub-nosed monkeys (*Rhinopithecus roxellana*) and in the rivers. We performed collinearity analysis between the high-risk ARGs (Rank I ARGs) from golden snub-nosed monkeys and the metagenome-assembled genomes (MAGs) from river samples to assess the potential of river microorganisms to acquire Rank I ARGs. The number of collinear events between Rank I ARGs and MAGs in the WHD group (rivers within the disturbed monkey habitat) was five times higher than that in the WCK (rivers within the undisturbed monkey habitat) group. This implies a significant spillover risk of ARGs from wild animals to the human ecosystem via river interfaces, a risk that is further intensified by human disturbances. This research complemented the content that evaluated the spillover risk of ARGs from wild animals to humans.

## Highlights

• HDs increased the spillover risk of ARGs in wild animals
• HDs could boost Rank Ⅰ ARG acquisition from wild animals by environmental microbes
• An omics approach to assess ARGs spillover from wild animals gut microbiota

## Graphical abstract

**Figure:**
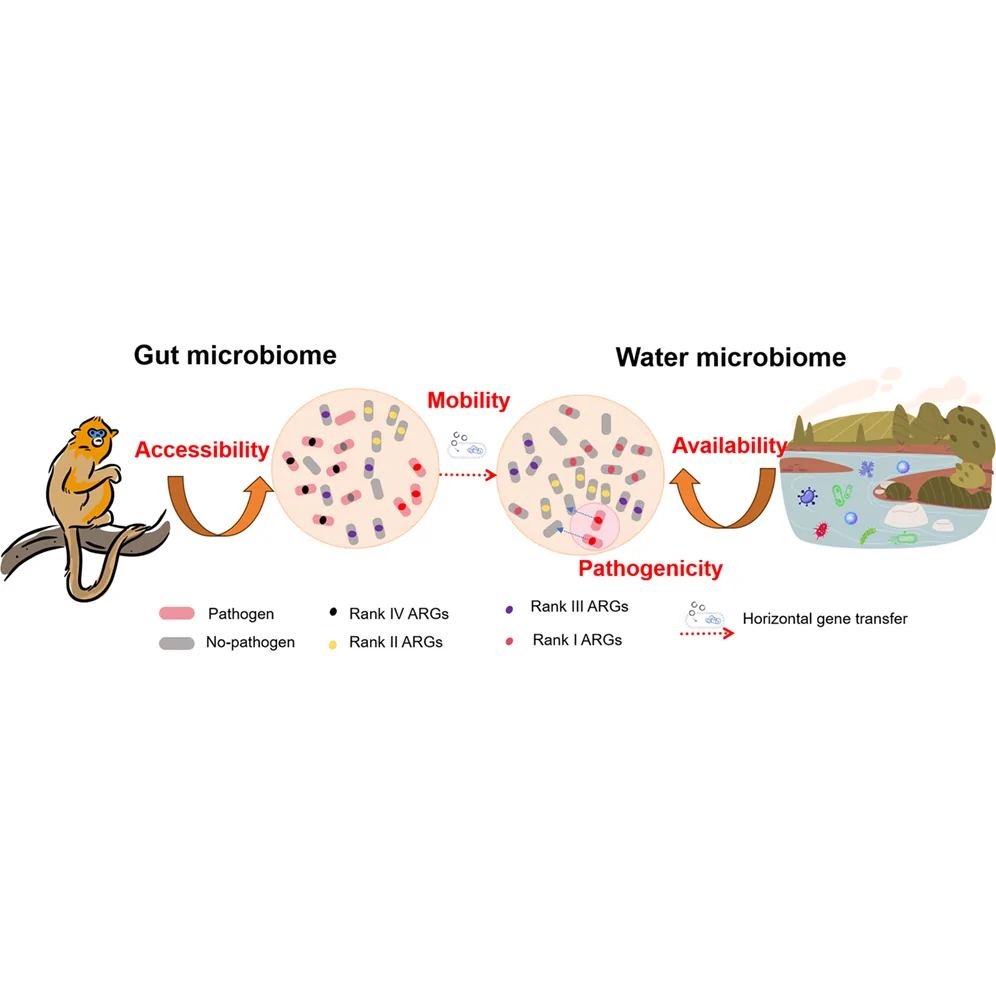

## Teaser

Biological sciences; Molecular biology; Microbiology; Cell biology

## Introduction

In wildlife nature reserves, the habitats of wild animals notably overlapped with those of humans. Although substantial investment of resources had been spent on wild animal conservation and their habitat construction, there was formidable a challenge: the contradiction between human economic activities and the protection of wild animals remained in China.^1^ Human activities such as grazing, cultivation, planting, and domestic sewage were still important factors that were affecting wild animals and their habitats.^2^ Under the coexistence of humans and wild animals, the wild animals and their habitats were affected by human activities, while the wild animals also influenced human livelihoods and daily practices.^3^

There is a substantial threat to human health and the ecological environment from antibiotic resistance genes (ARGs) and pathogenic antibiotic-resistant bacteria (PARBs) that spread, transfer, and diffuse in ecosystems.^4^^,^^5^^,^^6^ Human production activities such as animal husbandry, aquaculture, and plant agriculture widely use antibiotics, so wild animals could easily obtain ARGs and PARBs from these activities.^7^^,^^8^^,^^9^ Meanwhile, wild animals could serve as reservoirs of ARGs and PARBs, and spread them to humans and other animals.^10^^,^^11^ The realm of wildlife constitutes a distinct ecosystem where a substantial reservoir of pathogenic bacteria is naturally present. The introduction of ARGs under human disturbances (HDs) may drive unintended co-evolution of ARGs and virulence factors (VFs) in wild animal pathogens. More seriously, many studies have shown that the stress of HDs would increase the abundance and mobility of ARGs in the gut microbiome of wild animals.^1^^,^^4^^,^^11^ Therefore, whether directly or indirectly, wild animals play an important role in the spread of ARGs.^12^ Frequent contact between humans and wildlife may elevate the risk of pathogenic ARGs spilling over from wild animals to humans.

Not all ARGs pose health risks to public health, highlighting the importance of identifying those at high risk.^11^ A framework to assess the ecological risk of ARGs had been designed that was established based on three criteria: human accessibility, gene mobility, and host pathogenicity.^13^ Currently, high-risk ARGs are used to directly evaluate the risks that they pose to public health. After high-risk ARGs are released into the public environment, they might be acquired by environmental microorganisms and insert into their microbial genomes in order to be stably inherited and pose continuous risks; this process is the tolerance or availability of environmental microorganisms to exogenous ARGs. The efficiency of ARG integration into microorganisms is hindered by barriers imposed by both recipient specificity and various environmental factors.^1^ However, previous studies have generally focused on predicting the spillover risk of ARGs from wildlife based on their characteristics,^4^ with little attention to the potential of environmental microorganisms to acquire these high-risk ARGs.

There are many types of wild animals, but their ecological threat types are different. Non-human primates are important model animals for biological research. It is more suitable for application in the field of interaction between humans and wild animals, enabling studies on the behaviors of such interactions, ecological behaviors,^14^ and the “One Health”.^11^ The golden snub-nosed monkeys belong to the category of non-human primates, and they are one of the flagship species for wild animals’ conservation in China. We have demonstrated that the risks of ARGs in the gut microbiome of golden snub-nosed monkeys were increased by HDs; their host microorganisms were published pathogens, which also carried mobile genetic elements (MGEs).^1^^,^^4^ Research has confirmed that the environment in habitats could affect the exposure of ARGs from wild animals and their degree of diffusion in the ecosystem.^15^^,^^16^ ARGs originating from wild animals do not discharge directly into river systems but undergo intricate environmental processes prior to entry. In these complex ecological matrices, horizontal gene transfer constitutes a predominant risk factor for the propagation and persistence of ARGs.^17^^,^^18^ In nature, microorganisms primarily acquire new genetic information through three mechanisms: conjugation, transformation, and transduction; in all three processes, the exogenous DNA obtained by host microorganisms undergoes semi-conservative replication after pairing with homologous regions of the recipient bacterial DNA, thereby integrating into the microbial community of the aquatic environment.^19^^,^^20^ Rivers play an important role in the spread of ARGs and pathogenic antibiotic-resistant bacteria into ecosystems, because ARGs in the rivers could be directly exposed to humans through drinking, contact, or intake.^21^ The overlapping habitats between humans and wild animals promote the spread risks of ARGs and PARBs.^22^ Previous research studies have found that different contacts between humans and wild animals increased the types and abundance of ARBs in the river habitats of the golden snub-nosed monkeys.^2^ However, further research would be needed to identify which ARGs in the gut microbiome of the golden snub-nosed monkeys pose a spillover risk of spilling into the river ecosystem, and which microorganisms in the rivers could acquire these ARGs.

To tackle the above issues, we used omics techniques to obtain ARGs, MGEs, and VFs from the gut microbiome of golden snub-nosed monkeys and, according to the wild animals associated enrichment, ARG mobility and pathogenicity, to obtain the Rank I ARGs. Then, collinearity analysis of Rank I ARGs in the gut microbiome of the golden snub-nosed monkeys and metagenome-assembled genomes (MAGs) in the river habitats was conducted to investigate the microorganisms in rivers that may acquire Rank I ARGs and assessing their potential for Rank I ARG acquisition. Finally, we used structural equation modeling (SEM) to identify the influencing factors of Rank I ARGs spilled over from the golden snub-nosed monkeys to rivers. Identifying the characteristics of pathogenic ARGs from wild animals that come into contact with humans, analyzing their pathogenic mechanism, and determining the microbial species that harbor these ARGs, as well as predicting their spillover risk to humans, could enable risk assessment of the most likely sources of future epidemics.

## Results

### The risk levels of ARGs in the gut microbiome of golden snub-nosed monkeys

A total of 128,291 ARG-contigs were detected in the gut microbiome of the golden snub-nosed monkey, and they were divided into 21 classes of ARGs, which were all found in the HD group (golden snub-nosed monkeys with human disturbance) and W group (wild golden snub-nosed monkeys without human disturbance). In the HD group, the relative abundance of diaminopyrimidine resistance genes was significantly higher than that in the W group (*p* < 0.001) (Figure 1A). In addition, the relative abundances of resistance genes conferring resistance to fluoroquinolone (*p* = 0.037), multidrug (*p* = 0.037), beta-lactam (*p* = 0.037), phenicol (*p* = 0.037), and mupirocin (*p* = 0.038) were significantly higher in the HD group than in the W group.

**Figure 1:**
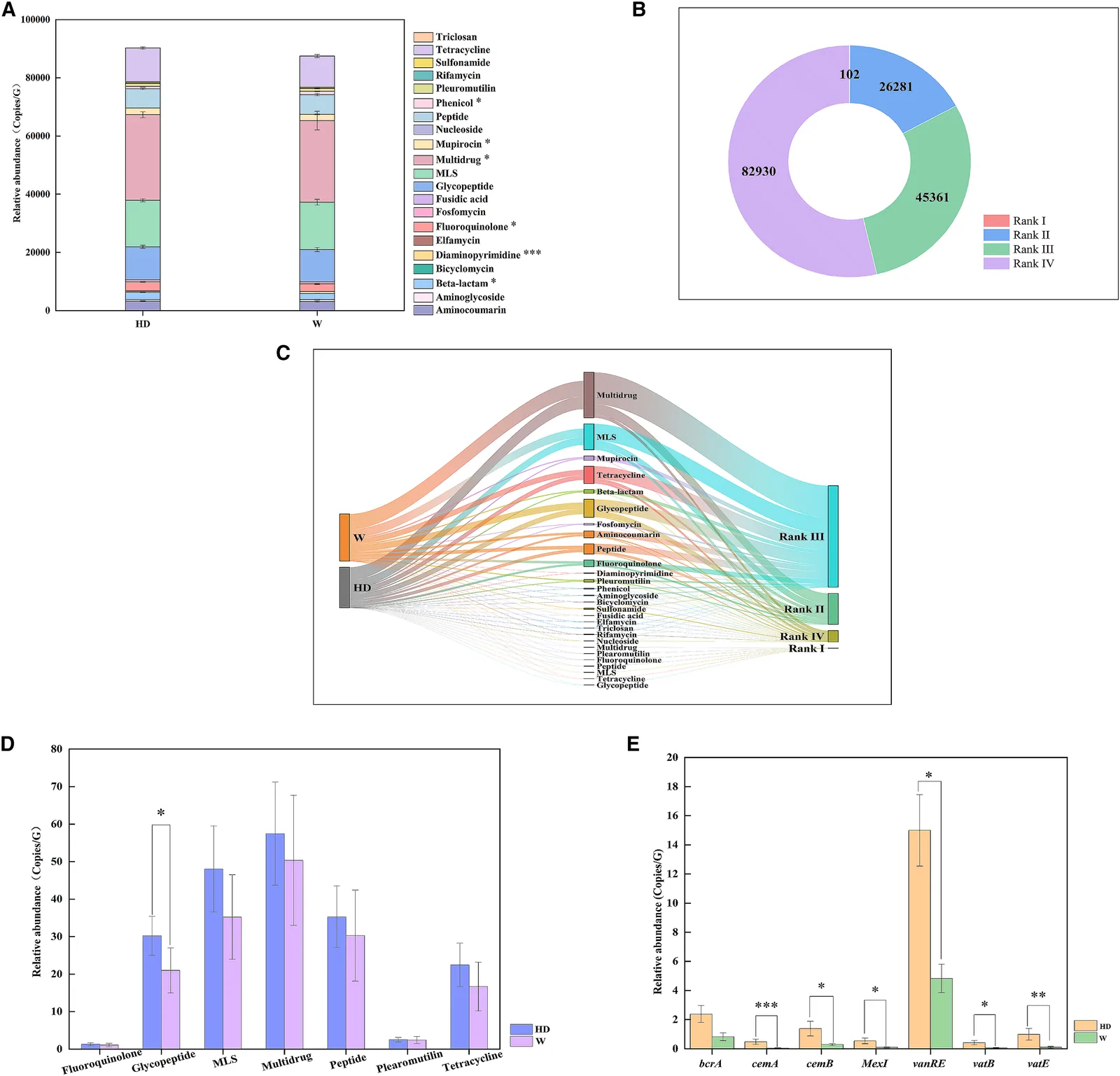
The characteristics of ARGs in the golden snub-nosed monkeys (A) The relative abundance of ARG classes is shown. (B and C) The number of ARGs-contigs and ARG classes in the golden snub-nosed monkeys are shown. (D and E) The relative abundance of ARG classes and the subtypes of Rank I ARGs are shown. The HD group referred to the golden snub-nosed monkeys with human disturbed, and the W group referred to wild golden snub-nosed monkeys without human disturbed; the same applies hereafter. Statistical analysis was performed with the Mann-Whitney U test (*n*_HD_ = 15, *n*_W_ = 15); error bars represented the mean ± SD; the *p* values were corrected using false discovery rate (FDR) (*q* = 0.05). ∗*p* < 0.05, ∗∗*p* < 0.01,∗∗∗*p* < 0.001.

In the gut of wild animals, Rank Ⅰ to Ⅳ ARGs are classified by their key traits: Rank Ⅰ are enriched, mobile, and pathogenic; Rank Ⅱ are enriched and mobile; Rank Ⅲ are accessible; and Rank Ⅳ are non-accessible. There were 82,930, 45,361, 26,281, and 102 ARG-contigs that were divided into Rank IV, Rank III, Rank II, and Rank I, respectively (Figure 1B). Among them, Rank IV contained 21 classes of ARGs, Rank III contained 20 classes of ARGs, Rank II contained 18 classes of ARGs, and Rank I contained only 7 classes of ARGs (Figure 1C).

Among the 7 classes of Rank I ARGs, glycopeptide resistance genes showed a significantly higher relative abundance in the HD group compared to the W group (*p* = 0.019) (Figure 1D). Besides, the 7 classes of Rank I ARGs were divided into 42 subtypes. The top seven subtypes in terms of relative abundance in the HD group were *bcrA*, *cemA*, *cemB*, *MexI*, *vanRE*, *vatB*, and *vatE*. Among them, except for bcrA, the relative abundances of *cemA* (*p* < 0.001), *vatE* (*p* = 0.007), *cemB* (*p* = 0.012), *vanRE* (*p* = 0.012), *MexI* (*p* = 0.030), and *vatB* (*p* = 0.036) were significantly higher in the HD group than in the W group (Figure 1E). Notably, *vanRE* is a subtype of glycopeptide resistance genes. The characteristics of Rank I ARGs are shown in Table S2.

### Characteristics of the microorganisms in rivers

A total of 95 MAGs were identified in the river samples. Among these, 20 were found exclusively in the WCK group (control rivers), 85 were found exclusively in the WHD group (rivers within disturbed golden snub-nosed monkeys’ habitats), with 10 MAGs shared between the 2 groups (Table S2). The relative abundances of each MAG in the WCK group and the WHD group were shown in Figure S1. The MAGs were classified into 5 phyla in the WCK group and 8 phyla in the WHD group. Among them, Bacillota, Bacteroidata, Methanobacteriota, and Verrucomicrobiota were found only in the WHD group. The relative abundance of Pseudomonadota (*p* = 0.048) was significantly higher in the WHD group than that in the WCK group, whereas that of Actinomycetota (*p* = 0.043) was significantly lower in the WHD group than that in the WCK group (Figure 2A).

**Figure 2:**
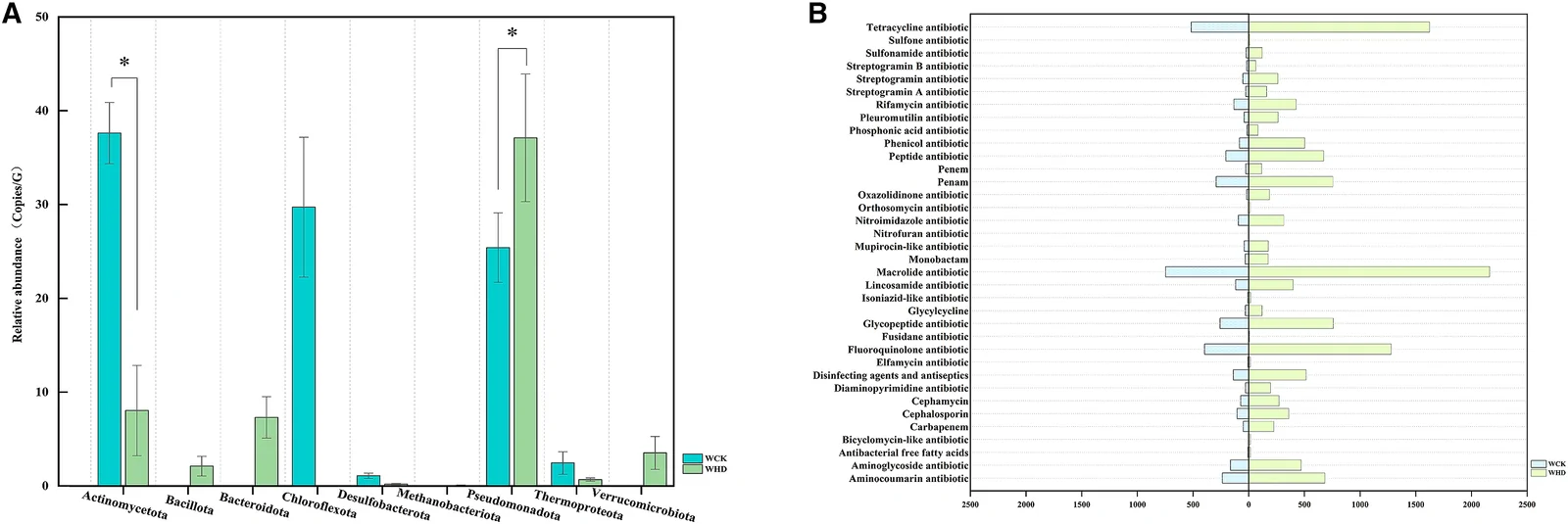
The characteristics of microorganisms in the rivers (A) The relative abundances of the microbiome in the WCK group and the WHD group are shown. (B) The numbers of ARGs in the WCK group and the WHD group are shown. The WHD group referred to rivers in disturbed golden snub-nosed monkey habitats, and the WCK group referred to the control rivers; the same applies hereafter. Statistical analysis was performed with the Mann-Whitney U test (*n*_WHD_ = 9, *n*_WCK_ = 9); error bars represented the mean ± SD; the *p* values were corrected using FDR (*q* = 0.05). ∗*p* < 0.05.

A total of 37 types of ARGs were identified in riverine microorganisms. The total number of ARGs in the WHD group was higher than that in the WCK group. The WHD group contained 13,426 ARGs, while the WCK group contained 3,996. Macrolide ARGs were the most abundant category in both groups, with 2,163 in the WHD group and 746 in the WCK group. Fusidic acid resistance genes and nucleoside ARGs were found exclusively in the WHD group, with counts of 7 and 11, respectively. In contrast, nitrofuran antibiotic and orthosomycin ARGs were only detected in the WCK group, each with a count of 3 (Figure 2B).

### The availability of Rank I ARGs by microorganisms in the rivers

There were 84 Rank I ARGs-contigs in the W group of golden snub-nosed monkeys that were collinear with 20 MAGs in the WCK group of the rivers 224 times, and there were 5 kinds of microorganisms in the WCK group that had the potential of being collinear with 5 kinds of bacteria in the gut microbiome of golden snub-nosed monkeys (Figure 3A). There were 102 Rank I ARGs-contigs in the HD group of golden snub-nosed monkeys that were collinear with 85 MAGs in the WHD group of the rivers 1,008 times, and there were 8 kinds of microorganisms in the WHD group that had the potential of being collinear with 6 kinds of bacteria in the gut microbiome of golden snub-nosed monkeys. Enterococcaceae were the new types of bacteria in the HD group that could carry Rank Ⅰ ARGs, and the Bacillota, Bacteroidata, Methanobacteriota, and Verrucomicrobiota were new types in the WHD group that could accept ARGs (Figure 3B). HDs significantly increased the total relative abundance of the host bacteria of Rank I ARGs (*p* = 0.014); the relative abundance of *Helicobacter* was the highest in the gut microbiome of the golden snub-nosed monkeys, and the relative abundance of *Campylobacter* was the second highest (Figure 3C).

**Figure 3:**
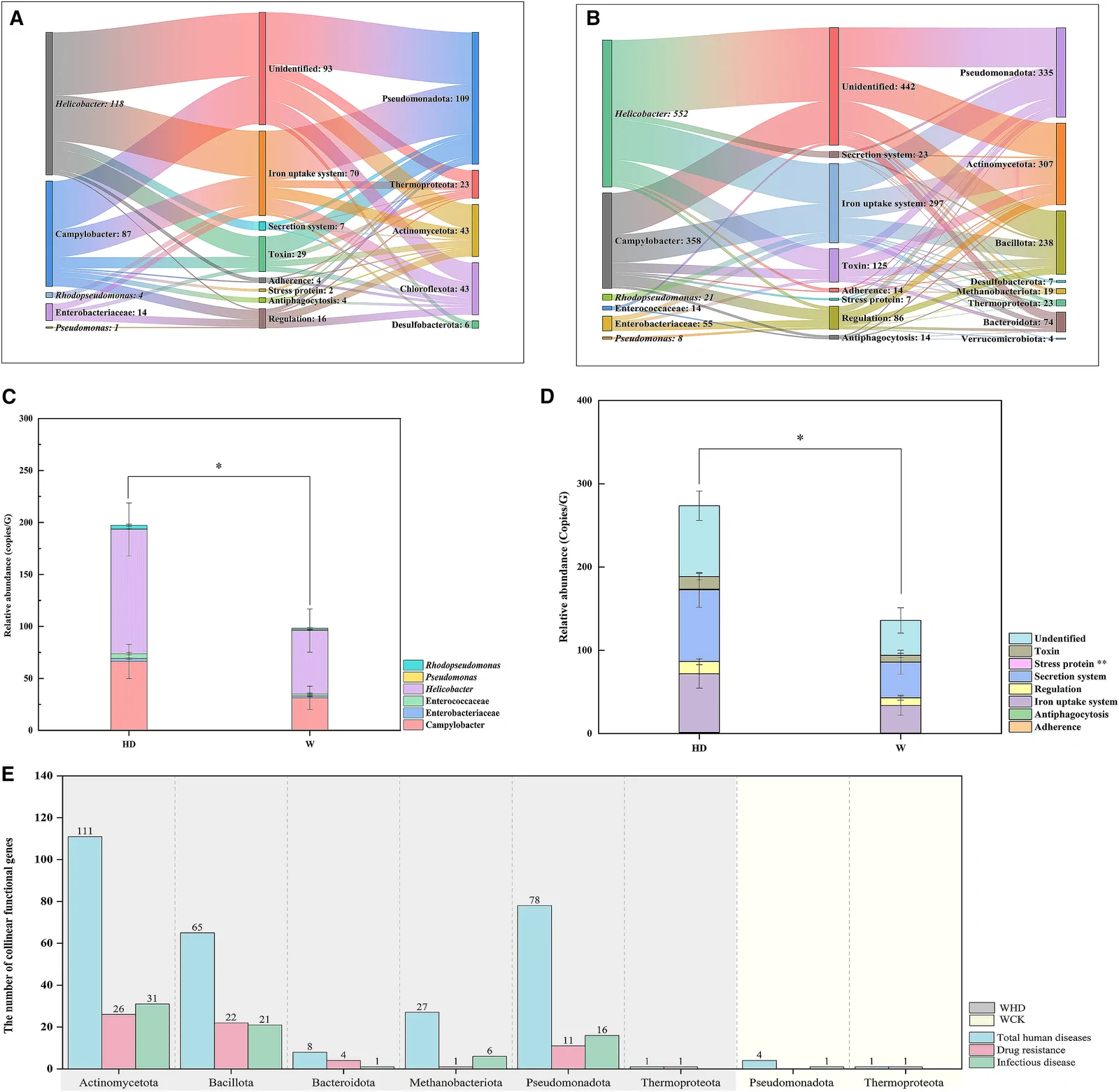
The pathogenicity from golden monkeys to rivers (A and B) The relationship that the Rank I ARGs were collinear with MAGs in the WCK group and the WHD group is shown. (C) The relative abundance of the host bacteria of the Rank I ARGs in golden snub-nosed monkeys is shown. (D) The relative abundance of VFs in Rank I ARGs of the golden snub-nosed monkey is shown. (E) The number of functional genes in microorganisms from the WHD and WCK groups that were collinear with human disease pathways. The numbers in (A) and (B) represented the number of collinearity events. Statistical analysis was performed with the Mann-Whitney U test (*n*_HD_ = 15, *n*_W_ = 15); error bars represented the mean ± SD; the *p* values were corrected using FDR (*q* = 0.05).∗*p* < 0.05, ∗∗*p* < 0.01.

There were 8 kinds of VFs in Rank I ARGs, and the total relative abundances of VFs were significantly increased under HDs (*p* = 0.016). Specifically, the relative abundance of the stress protein VF was significantly higher in the HD group than in the W group (*p* = 0.008). In Rank I ARGs, the relative abundance of unidentified VFs was the highest (Figure 3D).

In the WCK group, only 4 MAGs that were collinear with Rank I ARGs were found to contain functional genes associated with human disease pathways, while the WHD group contained 45 such MAGs (Table S3). In the microbiota of the WCK group, there were only 5 functional genes that were collinear with human disease pathways, whereas in the WHD group, this number reached as high as 290. Among them, the functional genes related to drug resistance and infectious disease under the secondary classification level are respectively 65 and 75 in the WHD group, while there is only 1 in the WCK group. The functional gene information of other collinear human disease pathways is shown in Table S3.

### The spillover mechanism of ARGs from wild animals to the rivers

In the gut microbiome of the golden snub-nosed monkeys, the total abundances of MGEs and the total abundances of spillover risk had a positive correlation, while the total abundances of MGEs and the food diversity had a negative correlation; however, not all of them were significant (Figure 4A). This indicated that the factors influencing the spillover risk of ARGs in the intestines of wild animals are complex.

**Figure 4:**
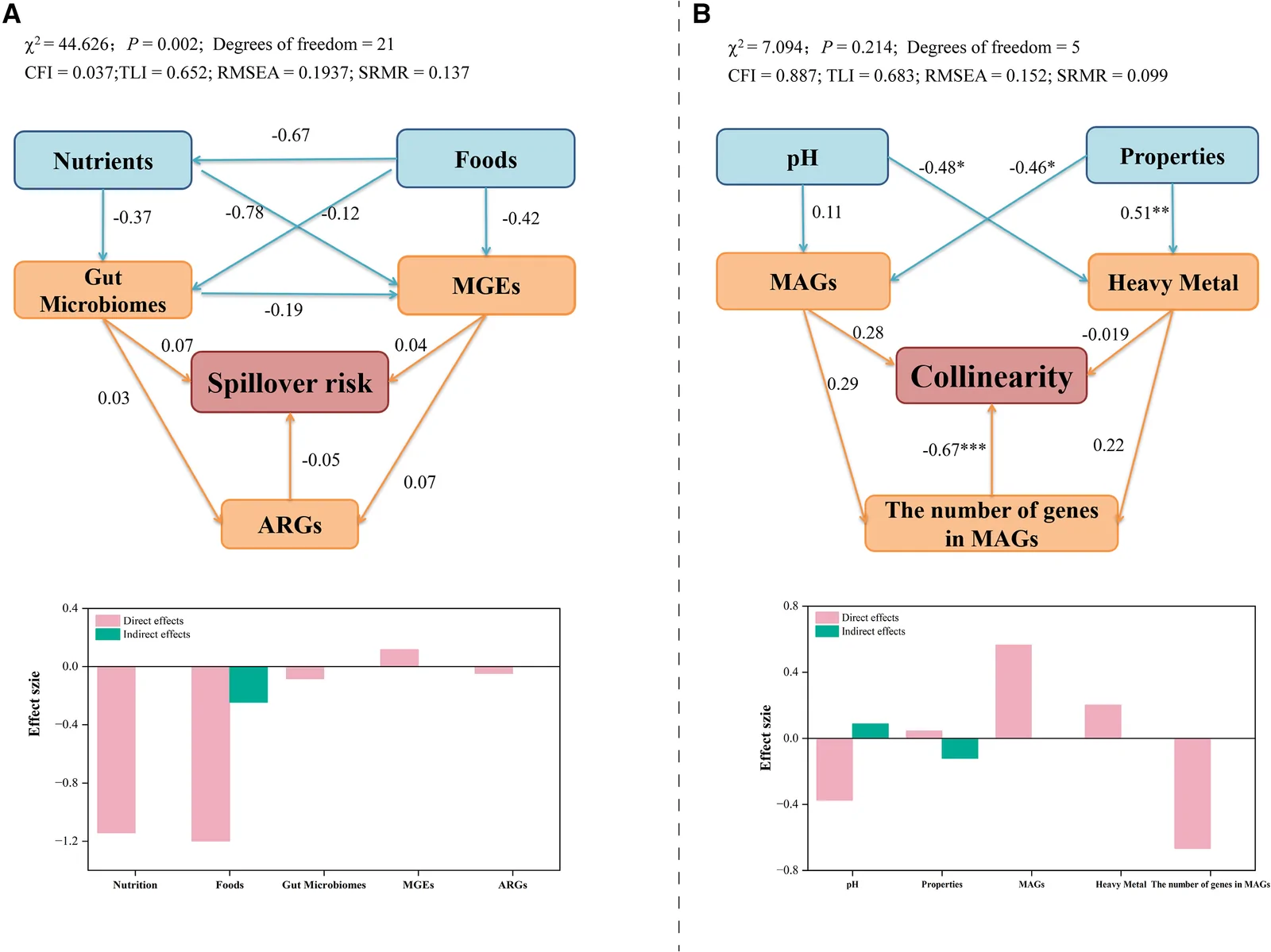
The spillover mechanism of ARGs from wild animals to the rivers (A) Representation of the influencing factors of the spillover risk of ARGs. (B) Representation of the influencing factors of the collinearity ability of river microorganisms. The structural equation model is estimated using the maximum likelihood method. ∗*p* < 0.05, ∗∗*p* < 0.01, ∗∗∗*p* < 0.001.

In the rivers, the gene numbers of MAGs were significantly negatively correlated with their collinearity ability (r = −0.67, *p* = 0.003). The relative abundance of MAGs showed a positive correlation with collinearity (r = 0.28), but not significant. The physicochemical properties of the rivers were significantly negatively correlated with the relative abundance of MAGs (r = −0.46, *p* = 0.032), and the physicochemical properties were significantly positively correlated with heavy metals (r = 0.51, *p* = 0.007). Meanwhile, the pH was significantly negatively correlated with heavy metals (r = −0.48, *p* = 0.011) (Figure 4B).

## Discussion

There were ARGs in wild animals, and the resistance risk in the gut microbiome of golden snub-nosed monkeys was increased because they came into contact with humans frequently.^11^^,^^23^ The gut microbiome resistance of golden snub-nosed monkeys had the potential to migrate to the human ecosystem under HDs.^4^^,^^24^ Fortunately, most of the detected ARGs appear to have limited potential for transmission to the environment or to humans. In our research, there were ARGs in the gut microbiome of golden snub-nosed monkeys, and the gut microbiome resistance of golden snub-nosed monkeys was enhanced by HDs. Rank I ARGs of golden snub-nosed monkeys had the fewest species and the lowest total abundance, which was supported by other research studies.^1^^,^^4^^,^^11^ Nonetheless, the potential risks of Rank I ARGs should be emphasized, as they represent a crucial determinant in the assessment of health risks.^13^ Overall analysis showed that the contact between humans and wild animals had increased the spillover risks of ARGs from wild animals to humans, because, under HDs, the relative abundance of host bacteria carrying Rank Ⅰ ARGs increased significantly, along with a notable rise in the relative abundance of glycopeptide resistance genes. The abundance of Rank I ARGs at the gene and subtype levels had increased. In particular, the relative abundance of the glycopeptide resistance gene subtype *VanRE* showed a highly significant increase. Our results were supported by Zou et al.^4^ Therefore, we recommend that within wildlife natural reserves, alongside preventing antibiotic misuse, monitoring for ARGs should be intensified, with targeted surveillance specifically for the glycopeptide antibiotic resistance gene *VanRE*. According to previous research, *Helicobacter* could cause a variety of gastric and extragastric diseases^25^^,^^26^ and *Campylobacter* could lead to the diseases of gastroenteritis,^27^ endocarditis, and meningitis, and so on. The Enterococcaceae could cause urinary tract infections,^28^ abdominal and pelvic infections, sepsis,^29^ and endocarditis,^30^ and so on. Undeniably, enhanced monitoring should be implemented for the pathogenic host bacteria of Rank I ARGs, including *Campylobacter*, *Helicobacter*, Enterobacteriaceae, Enterococcaceae, and *Pseudomonas*.

Under HDs, there were some new kinds of microorganisms, such as Bacillota, Bacteroidota, Methanobacteriota, and Verrucomicrobota, in the rivers, and the relative abundance of Pseudomonadota in the river habitats had been significantly increased; meanwhile, we detected higher ARG levels in disturbed rivers. These results were supported by some other studies.^2^^,^^31^^,^^32^ Besides, the higher the abundance and diversity of microorganisms, the stronger the overall function of microorganisms.^13^ This indicated that microorganisms in rivers under HDs might have an increased potential to acquire and integrate Rank I ARGs in the golden snub-nosed monkeys. Homologous recombination is also called general recombination. The direct exchange of homologous sequences between two DNA molecules is homologous recombination. Homologous recombination is the driving force for host bacteria to obtain new traits. Our research results found that all kinds of MAGs in the rivers had homology with Rank I ARGs in the gut microbiome of the golden snub-nosed monkeys, and their collinearity ability had been increased by HDs, which indicated that the potential of microorganisms in the rivers to acquire Rank I ARGs in the golden snub-nosed monkeys might be increased by HDs. These research results supported our hypothesis that microorganisms in the rivers were available for Rank I ARGs, and the Rank I ARGs had the potential to be stably inherited in the microorganisms in the rivers. Due to the fluidity of rivers, ARGs could rapidly transfer in the rivers or accumulate in bottom sludge. Thus, if the Rank I ARGs from wild animals enter rivers, they could directly pose a risk to humans in protected areas.^2^^,^^33^ In our study, all the MGEs carrying Rank I ARGs were plasmids. This indicates that the Rank I ARGs harbored by golden snub-nosed monkeys have the potential to be transferred to the microbiota in riverine environments, which could subsequently increase the pathogenic potential of the riverine microbiota. Some MAGs in the rivers that did not have pathogenic pathways also had the ability to integrate the Rank I ARGs from the golden snub-nosed monkeys, and this ability was increased by HDs. Furthermore, bacteriophages are critical mediators of ARG dissemination through transduction.^34^ Future work integrating viromic sequencing and prophage prediction is needed to systematically assess the role of phages in ARG dissemination.

Our research indicated that HDs increased the risk of Rank I ARGs that flowed to the human ecosystem; the research result was the same as the research of Zou et al.^11^ According to our research, there were several factors that led to this result. Firstly, accessibility and the frequency of collinearity increased. Under HDs, the number of collinear events between Rank I ARGs and MAGs in the WHD group was five times higher than that in the WCK group, potentially indicating that HDs have increased the spillover risk. Secondly, the potential of river microorganisms that carried Rank I ARGs was enhanced. Under HDs, the abundances and types of river microorganisms and Rank I ARGs could be captured with high probability by Pseudomonadota in the rivers. The host bacterium Enterobacteriaceae, which harbored Rank I ARGs collinear with the river microbial community, was only found in the HD group. Enterococcaceae had risks that could cause inflammation in humans.^35^ Thirdly, the ability to carry VFs was enhanced; the abundance of VFs was increased by HDs, which was supported by the research of Hu et al.^2^ The proportion of unclassified VFs was the largest; unclassified VFs might bring unknown health risks to public health. Fourthly, the ability of microbial acquisition was enhanced; the microbial types that could accept Rank I ARGs were increased by HDs, which was supported by the research of Zou et al.^4^ Thus, HDs increased the risk of Rank I ARGs flowing to human ecosystems by increasing accessibility, mobility, and pathogenicity of Rank I ARGs, and the ability to be acquired by microorganisms in the rivers. It is important to note that human interference has increased the number of collinear functional genes between Rank I ARGs and human disease pathways in river microorganisms, particularly in drug resistance and infectious diseases. Additionally, although bacteria of the genus *Rhodopseudomonas* are not pathogenic,^36^ golden snub-nosed monkeys may ingest them through activities such as drinking water and foraging, allowing them to temporarily pass through their intestines. As the animal gut serves as a complex microbial gene exchange pool, *Rhodopseudomonas* may acquire Rank I ARGs via MGEs, such as plasmids and transposons, from other pathogenic and drug-resistant bacteria present in the intestines of golden snub-nosed monkeys.^37^ Therefore, *Rhodopseudomonas* may serve as potential host bacteria for the transmission of Rank I ARGs. This also indicated that human activities increase the risk of Rank I ARGs spillover from the gut microbiota of wild animals.

In our study, we hypothesized that during the transfer of ARGs from wild animals to the human ecosystem, there were two processes involved: the first process was the spillover risk of ARGs in the gut microbiome of the wild animals. The second process was the microbial reception in the rivers. We assumed that, in the first process, the gut microbiome was the host of ARGs; the food and soluble nutrients affected the characteristics of the gut microbiome, and the MGEs could carry ARGs. However, the MGEs were influenced by the abundance of host bacteria; the higher the abundance of host bacteria, the stronger the ability of MGEs to move ARGs.^38^^,^^39^ We set that in the second process, when the HDs affected the physicochemical properties of the rivers, and through the properties of the rivers, affected the microbial characteristics and microbial gene quantity in the rivers, and then affected the potential of rivers microbiome to obtain Rank I ARGs.

Environmental factors are key drivers of microbial risks.^40^ In this study, nutrient content was negatively correlated with MAG abundance but positively correlated with heavy metal concentrations, whereas pH exhibited a negative correlation with heavy metal levels. In wildlife nature reserves, anthropogenic activities contribute to the concurrent input of nutrients and heavy metals. Low pH conditions can enhance the dissolution and bioavailability of heavy metals from sediments,^41^ thereby promoting the conversion of heavy metals from bound to exchangeable forms and increasing their effective availability.^42^ Heavy metal stress can enrich ARG-carrying resistant taxa through co-selection.^43^ Furthermore, low-nutrient conditions can promote the proliferation of specific microorganisms, reflecting an ecological strategy in which microorganisms enhance antibiotic resistance to gain a competitive advantage under nutrient-limited conditions.^44^ Therefore, heavy metals introduced by anthropogenic activities, under low-pH and low-nutrient conditions, could indirectly promote the persistence and dissemination of ARGs in MAGs by increasing heavy metal bioavailability and exerting selective pressure, thereby enhancing the acquisition potential of environmental MAGs for ARGs from the gut microbiome of golden snub-nosed monkeys and increasing the spillover risk. Besides, we also found that physicochemical properties in the rivers could positively affect the potential of MAGs in the rivers to accept Rank I ARGs by affecting the content of heavy metals, which was the same as other findings that physical and chemical properties have been identified as important environmental factors that promote the spread of ARGs.^45^ Meanwhile, MAGs with fewer genes in rivers could exhibit a stronger potential to acquire Rank I ARGs, likely because smaller genomes facilitate faster evolutionary adaptation.^46^ Finally, we suggested that in the natural reserve, the feeding of wild animals should be monitored and the discharge of domestic sewage and chemical sewage should be controlled.

### Limitations of the study

In this study, we observed that ARGs were enriched in the gut microbiome of golden snub-nosed monkeys under HDs, accompanied by enhanced pathogenicity of Rank I ARGs and an increased potential for their acquisition by riverine microbial communities. Our findings provide evidence that HDs can intensify the gut resistome of golden snub-nosed monkeys and that these ARGs may pose a spillover risk.

Due to the high susceptibility of antibiotics to degradation in the environment, residual antibiotic concentrations in environmental water bodies are typically at trace levels ranging from ng/L to μg/L. Moreover, in solid matrices such as soil and sediments, antibiotics can also exist in strongly bound forms with organic matter and mineral particles. Similarly, antibiotic residues in wildlife feces may be as low as the pg/g level.^47^^,^^48^ Furthermore, antibiotic exposure in wild animals is often intermittent, meaning single fecal sampling may miss peak exposure events; thus, repeated sampling is required to reflect true exposure levels. Microbial metabolism within the gut and chemical transformations during fecal storage can further lead to antibiotic degradation. Meanwhile, microorganisms themselves can also produce antibiotics. Consequently, there are numerous uncertainties associated with antibiotic concentration detection. However, without measuring antibiotic concentrations, research conclusions would remain confined to descriptive observations of ARG presence. Future studies should integrate antibiotic concentration measurements to more accurately assess the dynamic relationship between environmental selection pressure and the spread of resistance genes.

In addition, short collinear blocks are suitable for comparing closely related genomes and can identify rearrangements and collinearity. However, they may arise from random events or conserved sequences and do not necessarily represent genuine functional gene acquisition, so their inferential power is limited.

## Resource availability

### Lead contact

Requests for further information and resources should be directed to and will be fulfilled by the lead contact, Dayong Li (980119lsc@163.com).

### Materials availability

This study did not generate new, unique reagents.

### Data and code availability

• Metagenomic sequencing data have been deposited in the NCBI Sequence Read Archive (SRA) under the following accession numbers: SRA: PRJNA1330253 (river sediment samples) and SRA: PRJNA947945 (golden snub-nosed monkey fecal samples). The data can be accessed via the following links: https://www.ncbi.nlm.nih.gov/search/all/?term=PRJNA1330253 and https://www.ncbi.nlm.nih.gov/bioproject/?term=PRJNA947945. They are publicly available as of the date of publication. Accession numbers are listed in the key resources table.
• All original code has been deposited at Zenodo: https://doi.org/10.5281/zenodo.22445082 and is publicly available as of the date of publication.
• Any additional information required to reanalyze the data reported in this paper is available from the lead contact upon request.

## Acknowledgments

We thank the workers in Baihe National Nature Reserve for their help with the fresh sample collection and thank all members of the lab for their support. This work was supported by the 10.13039/501100001809National Natural Science Foundation of China (grant/award no. 32270548 and no. 32200403), the Sichuan Science and Technology Program (grant/award no. 2024NSFSC1970).

## Author contributions

S.L., data curation, investigation, methodology, software, validation, writing – original draft, methodology, conceptualization; J.M., writing – review and editing, investigation, methodology, software, validation, conceptualization; H.Z., investigation, methodology, software, validation; S.Z., writing – review and editing, visualization, supervision, resources, funding acquisition, conceptualization; D.L., writing – review and editing, visualization, supervision, resources, funding acquisition, conceptualization.

## Declaration of interests

The authors declare no competing interests.

## STAR★Methods

### Key resources table

**Table:** 

REAGENT or RESOURCE	SOURCE	IDENTIFIER
**Critical commercial assays**		

E.Z.N.A.® Soil DNA Kit	Omega Bio-Tek, Georgia, USA	Cat# D5625-01
NEXTFLEX Rapid DNA-Seq Kit	Bioo Scientific, USA	Cat# 5144-02

**Deposited data**		

Raw sequencing data (monkey feces)	This paper	NCBI SRA: PRJNA947945 (https://www.ncbi.nlm.nih.gov/bioproject/?term=%20PRJNA947945)
Raw sequencing data (river sediments)	This paper	NCBI SRA: PRJNA1330253 (https://www.ncbi.nlm.nih.gov/bioproject/?term=%20PRJNA1330253)

**Software and algorithms**		

fastp (v 0.20.0)	Chen et al.^49^	https://github.com/OpenGene/fastp
BWA (v 0.7.17)	Li and Durbin.^50^	http://bio-bwa.sourceforge.net
MEGAHIT (v 1.13)	Li et al.^51^	https://github.com/voutcn/megahit
CD-HIT (v 4.6.1)	Fu et al.^52^	https://www.bioinformatics.org/cd-hit/
MetaBAT (v 2.12.1)	Kang et al.^53^	https://bitbucket.org/berkeleylab/metabat
CheckM (v 1.2.2)	Parks et al.^54^	https://github.com/Ecogenomics/CheckM/wiki
GTDB-Tk (v 2.3.0)	Chaumeil et al.^55^	https://gtdb.ecogenomic.org/
SPAdes (v 3.15.5)	Bankevich et al.^56^	https://github.com/ablab/spades
Mauve (v 2.0)	University of Wisconsin Madison	http://darlinglab.org/mauve/
IBM SPSS Statistics (v 27.0.1)	IBM Corp., Armonk, NY, USA	https://www.ibm.com/spss
R (v 4.4.1)	R Foundation for Statistical Computing	https://www.r-project.org/
OriginPro 2024	OriginLab Corp., Northampton, MA, USA	https://www.originlab.com/
Microsoft Office 2010 Excel	Microsoft Corp., Redmond, WA, USA	https://www.microsoft.com/microsoft-365
Microsoft Office PowerPoint 2010	Microsoft Corp., Redmond, WA, USA	https://www.microsoft.com/microsoft-365
ArcGIS 10.5	ESRI, Redlands, CA, USA	https://www.arcgis.com/
Adobe Photoshop	Adobe Inc., San Jose, CA, USA	https://www.adobe.com/photoshop
R scripts for statistical analysis and figure generation (Figure 4)	This paper	https://doi.org/10.5281/zenodo.22445082
CARD (v 4.0.0)	McMaster University	https://card.mcmaster.ca/
VFDB (v 6.0)	Institute of Pathogen Biology, Chinese Academy of Medical Sciences & Peking Union Medical College	https://www.mgc.ac.cn/VFs/
ICEberg (v 2.0)	Shanghai Jiao Tong University	https://bioinfo-mml.sjtu.edu.cn/ICEberg2/
INTEGRALL	University of Aveiro; Inserm	https://integrall.bio.ua.pt/
ISfinder	Université Toulouse III - Paul Sabatier	https://www.isfinder.biotoul.fr
NCBI RefSeq (Plasmid database)	NCBI	https://ftp.ncbi.nlm.nih.gov/refseq/release/plasmid
NCBI NR database	NCBI	https://ftp.ncbi.nlm.nih.gov/blast/db/FASTA/
NCBI NT database	NCBI	https://www.ncbi.nlm.nih.gov

**Other**		

Nanodrop ND1000	Thermo Fisher Scientific, USA	Cat# ND-1000
Covaris M220	Covaris, China	NA
NovaSeq 6000 platform	Illumina, San Diego, California, USA	RRID: SCR_016387

### Experimental model and study participant details

#### Golden snub-nosed monkeys (Rhinopithecus roxellana)

The study subjects were golden snub-nosed monkeys (*Rhinopithecus roxellana*) inhabiting the Baihe National Nature Reserve, China. Fecal samples were collected randomly from individuals of both sexes and various age classes. All fieldwork was conducted with the permission of the Baihe National Nature Reserve Administration and in full compliance with national wildlife protection laws and regulations. Notably, our previous research findings have been adopted by this reserve. This study involved only the collection of freshly deposited fecal samples and was entirely non-invasive, involving no capture, handling, or disturbance of the animals. The experimental protocol was reviewed and approved by the Animal Ethics Committee of China West Normal University (Approval No. cwnu2022D009), and all procedures were performed in accordance with relevant institutional and national guidelines for animal welfare and ethical practice.

### Method details

#### Selection of study areas and sample points

The Baihe National Nature Reserve is currently known to be the most representative with the largest population and the highest density of the golden snub-nosed monkeys.^57^ The Fourth National Giant Panda Survey revealed that the reserve was severely affected by human activities. Through the analysis of infrared camera data, we found that the trace points of human economic activities overlapped with the home ranges of the golden snub-nosed monkeys, which indicated that HDs had historically occurred in the Baihe National Nature Reserve, and HDs were still ongoing. Therefore, this study proposed to select monkeys that have been severely disturbed by human activities in Xiapingdi (HD group), Baihe National Nature Reserve, and rivers within the disturbed monkey habitat (WHD group) as the research subjects. As control checks, the wild monkey populations in undisturbed mountainous areas were designated as the W group, and their habitat rivers were designated as the WCK group. In addition, the two monkey groups inhabited areas with nearly identical climatic conditions. Figure S2 depicted the home ranges of HD group and W group, as well as rivers points sampling in their habitats.

#### Samples collection in the habitats

With the use of fecal collectors, 15 fecal samples were randomly taken from the HD group and the W group, respectively. The feces were collected at 9 am and 5 pm every day. The methods for collecting fecal samples were reported by Zou et al.^4^ In order to ensure the freshness of the collected feces, we discarded the feces of the golden snub-nosed monkeys which were found in the study areas for the first time. Human activities mainly include agriculture, grazing and domestic sewage, so we chose the river sediments that affected by agriculture, grazing and domestic sewage as WHD group. Then 9 samples of WHD and 9 samples of WCK were collected, respectively. The column samplers were used to collect the top 10 centimeters of sediments. To minimize the influence of geographic patterns, we set up 3 replicates for the sampling points to minimize sampling error.^2^ Collected samples were immediately transported on dry ice to Meiji Biological Medicine Co., Ltd., Shanghai, China, and subsequently stored at −80°C for DNA high-throughput sequencing analysis.

#### Metagenomic sequencing

A 0.5 g aliquot was taken from each of the fresh golden monkey fecal samples in the HD and W groups, as well as from the WCK and WHD river sediment samples. Total DNA was extracted using the E.Z.N.A.® Soil DNA Kit (Omega Bio-Tek, Georgia, USA), with a negative control included during the extraction process. After DNA extraction, the quality and concentration of DNA were assessed using spectrophotometry (Nanodrop ND1000, USA) and 1.0% agarose gel electrophoresis. The isolated DNA was stored at −80°C for further analysis. Subsequently, the DNA was fragmented to approximately 400 bp using a Covaris M220 (Limitation, China), and paired-end (PE) libraries were constructed with the NEXTFLEX Rapid DNA-Seq Kit (Bioo Scientific, USA). The Paired-End (PE150) metagenomic sequencing was performed on the NovaSeq 6000 platform (Illumina, San Diego, California, USA) at Meiji Biological Medicine Co., Ltd., Shanghai, China. The data of fecal samples were deposited into the NCBI Sequence Read Archive (SRA) database under accession number PRJNA947945. The data of river sediments were deposited into the NCBI Sequence Read Archive (SRA) database under accession number PRJNA1330253.

High-quality sequences were obtained by trimming adapter sequences from the 3' and 5 ends of reads using fastp^49^ (https://github.com/OpenGene/fastp, version 0.20.0), and subsequently discarding reads shorter than 50 bp and with an average base quality score below Q20. Reads were then aligned against the host DNA sequences using BWA^50^ (http://bio-bwa.sourceforge.net, version 0.7.17), and those with high similarity were removed as host-derived contamination. The statistical table for each sample sequence after quality control was presented in Table S1.

#### Bioinformatic analysis of fecal samples from the golden snub-nosed monkey

High-quality reads were assembled into contigs using MEGAHIT.^51^ Contigs with a minimum length exceeding 300 bp were retained. Open reading frames (ORFs) were predicted from these retained contigs (read length ≥ 100 bp) using MetaGene. The per-base coverage depth for all contigs was calculated by mapping the original reads from each sample. Predicted gene sequences from all samples were clustered using CD-HIT^52^ (https://www.bioinformatics.org/cd-hit/,v 4.6.1; identity > 90%, coverage > 90%), and the longest gene within each cluster was selected as the representative sequence to construct a non-redundant gene catalog.

The non-redundant gene catalog generated from ORF prediction during metagenomic sequencing analysis was aligned against several databases to obtain specific data. ARGs were identified by comparison against the Comprehensive Antibiotic Resistance Database (CARD, https://card.mcmaster.ca/, v 4.0.0; BLAST, e-value ≤ 1e-5; accessed March 2025). Virulence factors (VFs) types and counts were predicted by alignment with the Virulence Factors Database (VFDB, https://www.mgc.ac.cn/VFs/, v 6.0; BLAST, e-value ≤ 1e-5; accessed March 2025). To predict Mobile Genetic Elements (MGEs), alignments were performed against the following databases: the Plasmid database (NCBI RefSeq, https://ftp.ncbi.nlm.nih.gov/refseq/release/plasmid; BLAST, e-value ≤ 1e-5; accessed April 2025), the Conjugative Element database (ICEberg, https://bioinfo-mml.sjtu.edu.cn/ICEberg2/index.php, v 2.0; BLAST, e-value ≤ 1e-5; accessed April 2025), the Integron database (INTEGRALL, http://integrall.bio.ua.pt/; BLAST, e-value ≤ 1e-5; accessed April 2025), and the Insertion Sequence database (ISfinder, https://www.isfinder.biotoul.fr; BLAST, e-value ≤ 1e-5; accessed April 2025). Additionally, the gene catalog was aligned against the Non-Redundant protein database (NR, https://ftp.ncbi.nlm.nih.gov/blast/db/FASTA/; BLASTp, e-value ≤ 1e-5; accessed April 2025) to obtain taxonomic classification information at various levels. Finally, taxonomic information for plants was obtained by aligning sequences against the Non-redundant Nucleotide database (NT, https://www.ncbi.nlm.nih.gov; BLASTp, e-value ≤ 1e-5; accessed April 2025). The calculation of relative abundance involves normalizing the coverage of predicted ORFs against ARGs, VFs, MEGs, NR, and NT for each sample based on the data volume (copies/Gb). The calculation formula is as follows:

$$Realative\;abundance=\sum_{1}^{n}\frac{N\times 150/L}{G}$$

Where n is the total number of ORFs within a sample, N is the number of high-quality reads that map to the ORFs, 150 refers to the length of the high-quality reads, L indicates the length of the target ORF, and G corresponds to the data volume (in Gb) of high-quality reads per sample.

#### The assembly of metagenome-assembled genomes (MAGs) from river sediment samples

Filtered high-quality reads were grouped by sampling categories (WCK and WHD) and co-assembled using MEGAHIT^51^ (https://github.com/voutcn/megahit, v 1.13) with default parameters. The resulting contigs were subsequently clustered using MetaBAT^53^ (https://bitbucket.org/berkeleylab/metabat, v 2.12.1) to obtain metagenome-assembled genomes (MAGs) (contig length ≥ 1000 bp). The obtained MAGs were assessed for quality using CheckM^54^ (https://github.com/Ecogenomics/CheckM/wiki). Subsequently, a refinement step was performed via the built-in refinement module in MetaWRAP to generate high-quality MAGs ( completeness≥ 50% and contamination≤ 10%). Throughout this pipeline, all metagenomic assemblies were dereplicated to remove redundant results. Taxonomic classification was then performed on the refined genomes using GTDB-Tk (v 2.3.0) based on the Genome Taxonomy Database.^55^ High-quality genome MAGs were subsequently reassembled using SPAdes^56^ to reconstruct MAGs. The assembled MAG sequences were then aligned against the KEGG database (https://www.genome.jp/kegg, v 108.0; BLASTp, e-value ≤ 1e-5, identity > 70%; accessed March 2025).

The relative abundance of each MAG was calculated by normalizing the number of reads mapped to that genome (based on average coverage) against the total reads of the sample. The calculation formula is as follows:

$$A_{i}=C_{i}\times \left(N_{i}\;/\;N\right)$$

Where Ai, Ci, and Ni represent the relative abundance, coverage, and the number of contigs within the i-th MAG, respectively. N denotes the total number of reads in the sample.

#### Spillover risk assessment of ARGs in the gut microbiome of the golden snub-nosed monkeys

Since horizontal gene transfer between microorganisms primarily occurs through the exchange of genetic fragments, we classified the risk levels of ARGs based on ARG-contigs and performed collinearity analysis with river microbial MAGs.

Although a dedicated database for pathogenic antibiotic-resistant bacteria in wildlife is currently unavailable, non-human primates serve as important model animals in biomedical research due to the convergence of gut pathogenic microbiota between humans and non-human primates. Therefore, the ARG risk assessment approach established by Zhang et al.^13^ for humans is applicable to this study. The first step in the assessment was to determine the risk level of ARGs in the gut microbiome of the golden snub-nosed monkeys. We developed the framework by categorizing the risk level of ARGs according to three aspects: (1) The ARGs with high abundance (enrichment ≥ 100); (2) ARGs were mobile; (3) Presence in pathogens. There were four risk categories of ARGs were classified: (1) Rank IV (lowest risk): ARGs that do not meet the first criterion; (2) Rank III: those that met the first, but not meet the second; (3) Rank II: those that met the first and second but not meet the third; (4) Rank I (highest risk): those that met all three criteria.^13^

The second step of the evaluation was to determine the availability of MAGs in the rivers to Rank I ARGs in the golden snub-nosed monkeys. The genes of scaffolds of MAGs in the rivers and the genes of non-redundant contigs in the golden snub-nosed monkeys were carried out for collinearity analysis. Mauve (v 2.0 ) software (a match seed weight of 15, a minimum locally collinear block length of 50 bp^58^)was used to analyze the collinearity. If there were collinear between MAGs in the rivers and Rank I ARGs in the golden snub-nosed monkeys, the MAGs in the river could have the availability to Rank I ARGs. The spillover risk of Rank I ARGs from wild animals’ gut microbiome was assessed based on their relative abundance. The framework for assessing the spillover risk of ARGs from wild animal gut microbiome to habitat rivers under human disturbance showed in Figure S3.

#### Measurements of environmental factors

Environmental factor measurements of the rivers were the same as our the research we had published.^2^ The pH of river sediment samples was measured using a pH meter (PHS-3C, China). Water content was determined via the gravimetric method, protein content via the Kjeldahl method, and soluble sugar and total sugar contents via the anthrone-sulfuric acid method. These parameters were used to reflect the nutritional status of golden snub-nosed monkeys.

Mercury (Hg), arsenic (As), and lead (Pb) were determined using atomic fluorescence spectrometry. For Hg and As, samples were digested with a nitric acid–hydrochloric acid mixture in a boiling water bath and then reduced with potassium borohydride (KBH_4_) for fluorescence measurement (with thiourea added for As prior to reduction). For Pb, samples were fully digested with a hydrochloric acid–nitric acid–hydrofluoric acid–perchloric acid mixture, followed by reduction with KBH_4_ for fluorescence determination.^59^ Cadmium (Cd) was analyzed by flame atomic absorption spectrometry following KI-MIBK extraction. After full acid digestion, the sample was treated in an approximately 1% hydrochloric acid medium with KI, extracted with methyl isobutyl ketone (MIBK), and the organic phase was aspirated into the flame for absorbance measurement at 228.8 nm.^60^ Chromium (Cr) was determined by flame atomic absorption spectrometry. Following complete acid digestion, the digestate was aspirated into a fuel-rich air–acetylene flame, and absorbance was measured at 357.9 nm.^61^ All results are expressed on a dry weight basis (mg/kg).

### Quantification and statistical analysis

In our analysis, we used Office 2010 excel to count data preliminarily. Statistical differences in ARGs and MAGs were assessed using the Mann-Whitney U test in IBM SPSS Statistics 27.0.1, with multiple testing corrected by the Benjamini–Hochberg FDR method and significance set at adjusted *p* < 0.05. ∗, ∗∗, and ∗∗∗ indicate adjusted *p* < 0.05, *p* < 0.01, and *p* < 0.001, respectively. In R 4.4.1, structural equation modeling (SEM) was conducted using the lavaan package, with model parameters estimated via the maximum likelihood method. Histograms and sankey diagram were perfromed using OriginPro 2024. The ARGs risk assessment framework was constructed using Microsoft Office PowerPoint 2010. Mapping with ArcGIS 10.5 and Photoshop.

## Supplemental information

Document S1. Figures S1–S3 and Table S1

Table S2. The characteristics of Rank Ⅰ ARGs in the gut of golden snub-nosed monkeys

Table S3. The number of functional genes in the human disease pathway that were collinear between gut Rank I ARGs of golden snub-nosed monkeys and river MAGs

## References

[1] Zou S., Lu T., Huang C., Wang J., Li D. (2023). Grazing disturbance increased the mobility, pathogenicity and host microbial species of antibiotic resistance genes, and multidrug resistance genes posed the highest risk in the habitats of wild animals. Front. Environ. Sci..

[2] Hu R., Ren M., Liang S., Zou S., Li D. (2024). Effects of antibiotic resistance genes on health risks of rivers in habitat of wild animals under human disturbance – based on analysis of antibiotic resistance genes and virulence factors in microbes of river sediments. Ecol. Evol..

[3] Qi M. (2024).

[4] Zou S., Yuan T., Lu T., Yan J., Kang D., Li D. (2023). Human Disturbance Increases Health Risks to Golden Snub-Nosed Monkeys and the Transfer Risk of Pathogenic Antibiotic-Resistant Bacteria from Golden Snub-Nosed Monkeys to Humans. Animals.

[5] Wu Q., Li A., Lin F. (2026). Pollution status and transmission risks of bacterialantibiotic resistance in plastisphere of aquatic environments. Environ. Sci..

[6] Zhang T., Fan L., Zhang Y.N. (2025). Antibiotic resistance genes in aquatic systems: Sources, transmission, and risks. Aquat. Toxicol..

[7] Yang X., Wang S., Tong C. (2025). Research progress on horizontal transfer of antibiotic resistance genes from livestock and poultry manure. Acta Vet. Zootech. Sin..

[8] Li D., Wang X., Zhang G., Wang Z., Huang K. (2022). Advances on antibiotic resistance genes (ARGs) in aquaculture environment. South China Fish. Sci..

[9] Sheng H., Wang F., Xing L., Fu Y., Wang Z., Xu M., Mei Z., Liu Y., Dou Q., Jiang X. (2023). Research advances on environmental behavior and control of antibiotic resistance genes in soil. Acta Pedol. Sin..

[10] Wu J., Huang Y., Rao D., Zhang Y., Yang K. (2018). Evidence for Environmental Dissemination of Antibiotic Resistance Mediated by Wild Birds. Front. Microbiol..

[11] Zou S., Hu R., Liang S., Lu T., Kang D., Li D. (2024). Assessment of health risk of antibiotics resistance genes from human disturbed habitat to wild animals: Metagenomic insights into availability and functional changes of gut microbiome. Ecotoxicol. Environ. Saf..

[12] Łopucki R., Stępień-Pyśniak D., Christensen H., Kubiński K., Lenarczyk E., Martinez-de-Tejada G., Kitowski I., Masłyk M. (2024). Interspecies transmission of antimicrobial-resistant bacteria between wild birds and mammals in urban environment. Vet. Microbiol..

[13] Zhang A.N., Gaston J.M., Dai C.L., Zhao S., Poyet M., Groussin M., Yin X., Li L.G., Van Loosdrecht M.C.M., Topp E. (2021). An omics-based framework for assessing the health risk of antimicrobial resistance genes. Nat. Commun..

[14] Sun T., Niu W., Li Z., Zhou Q., Huang C. (2024). Advances in function morpho;ogy of locomotion in non-human primates. J. Guangxi Norm. Univ..

[15] Marion V., Sylvain G., Franck P., Patrick D., Lionel B., Nicolas R., Audrey A., Salim A., Helene J.P., Michel G.C. (2016). Antimicrobial resistance in wildlife. J. Appl. Ecol..

[16] Swift B.M.C., Bennett M., Waller K., Dodd C., Murray A., Gomes R.L., Humphreys B., Hobman J.L., Jones M.A., Whitlock S.E. (2019). Anthropogenic environmental drivers of antimicrobial resistance in wildlife. Sci. Total Environ..

[17] Zhang B., Qin S., Guan X., Jiang K., Jiang M., Liu F. (2021). Distribution of Antibiotic Resistance Genes in Karst River and Its Ecological Risk. Water Res..

[18] Zhao R., Yu K., Zhang J., Zhang G., Huang J., Ma L., Deng C., Li X., Li B. (2020). Deciphering the mobility and bacterial hosts of antibiotic resistance genes under antibiotic selection pressure by metagenomic assembly and binning approaches. Water Res..

[19] Nieto-Claudin A., Deem S.L., Rodríguez C., Cano S., Moity N., Cabrera F., Esperón F. (2021). Antimicrobial resistance in Galapagos tortoises as an indicator of the growing human footprint. Environ. Pollut..

[20] Weisberg A.J., Chang J.H. (2023). Mobile Genetic Element Flexibility as an Underlying Principle to Bacterial Evolution. Annu. Rev. Microbiol..

[21] Yang J., Xu Z., Wan D., Wang X., Zhang X., Zhu Y., Guo J. (2024). Pollution characteristics of heavy metals, antibiotic and antibiotic resistance genes in the crested ibis and their habitat across different lifestyle and geography. Environ. Res..

[22] Dias D., Torres R.T., Kronvall G., Fonseca C., Mendo S., Caetano T. (2015). Assessment of antibiotic resistance of Escherichia coli isolates and screening of Salmonella spp. in wild ungulates from Portugal. Res. Microbiol..

[23] Sacristán I., Esperón F., Acuña F., Aguilar E., García S., López M.J., Cevidanes A., Neves E., Cabello J., Hidalgo-Hermoso E. (2020). Antibiotic resistance genes as landscape anthropization indicators: Using a wild felid as sentinel in Chile. Sci. Total Environ..

[24] Ahlstrom C.A., van Toor M.L., Woksepp H., Chandler J.C., Reed J.A., Reeves A.B., Waldenström J., Franklin A.B., Douglas D.C., Bonnedahl J., Ramey A.M. (2021). Evidence for continental-scale dispersal of antimicrobial resistant bacteria by landfill-foraging gulls. Sci. Total Environ..

[25] Zhu T.T., Wang L., Wang H.L., He Y., Ma X., Fan J.M. (2016). Helicobacter pylori participates in the pathogenesis of IgA nephropathy. Ren. Fail..

[26] Zulueta A., Caretti A., Signorelli P., Ghidoni R. (2015). Resveratrol: A potential challenger against gastric cancer. World J. Gastroenterol..

[27] Zou L., Li Y., Zhou G.L., Zhen B.J., Zhang P., Jiang N., Zhao F.L., Wang J.G., Li H.J., Wang Y.C. (2020). Laboratory investigation for one gastroenteritis outbreak caused by Campylobacter jejuni. Zhonghua Liuxingbingxue Zazhi.

[28] Zhu Z., Du W., Yang Y., Zhang Y., Feng J., Wang Y. (2025). Enterococci independently increase the risk for initial antibiotic treatment failure and prolonged hospitalization in adult patients with complicated urinary tract infection: a retrospective cohort study. Infection.

[29] Ye J.J., Shie S.S., Cheng C.W., Yang J.H., Huang P.Y., Wu T.S., Lee M.H., Huang C.T. (2018). Clinical characteristics and treatment outcomes of vancomycin-resistant Enterococcus faecium bacteremia. J. Microbiol. Immunol. Infect..

[30] Zimmermann V., Fourré N., Ledergerber B., Epprecht J., Siedentop B., Monney P., Tzimas G., Frank M., Senn L., Ianculescu N. (2025). Evaluation of Different Versions of the Duke Criteria for the Diagnosis of Infective Endocarditis Among Patients With Enterococcal Bacteremia; a Multicenter Study. Open Forum Infect. Dis..

[31] Xing Y. (2023).

[32] Zhang H.N., Cui N., Shen H.M. (2021). Metagenomic Analysis Provides Insights into Bacterial Communities, Antibiotic Resistomes, and Public Health Risks in the Dongping Lake Reservoir. Huanjing Kexue.

[33] Xiong W. (2017).

[34] Liao H., Liu C., Zhou S., Liu C., Eldridge D.J., Ai C., Wilhelm S.W., Singh B.K., Liang X., Radosevich M. (2024). Prophage-encoded antibiotic resistance genes are enriched in human-impacted environments. Nat. Commun..

[35] Lotz H., Strahm C., Zdravkovic V., Jost B., Albrich W.C. (2019). Septic arthritis due to streptococci and enterococci in native joints: a 13 year retrospective study. Infection.

[36] Liu X., Huang L., Rensing C., Ye J., Nealson K.H., Zhou S. (2021). Syntrophic interspecies electron transfer drives carbon fixation and growth by Rhodopseudomonas palustris under dark, anoxic conditions. Sci. Adv..

[37] Lerner A., Matthias T., Aminov R. (2017). Potential Effects of Horizontal Gene Exchange in the Human Gut. Front. Immunol..

[38] Lu L., He Y., Peng C., Wen X., Ye Y., Ren D., Tang Y., Zhu D. (2022). Dispersal of antibiotic resistance genes in an agricultural influenced multi-branch river network. Sci. Total Environ..

[39] Forsberg K.J., Reyes A., Wang B., Selleck E.M., Sommer M.O.A., Dantas G. (2012). The shared antibiotic resistome of soil bacteria and human pathogens. Science.

[40] Chen H., Liu C., Teng Y., Zhang Z., Chen Y., Yang Y. (2021). Environmental risk characterization and ecological process determination of bacterial antibiotic resistome in lake sediments. Environ. Int..

[41] Wang Y., Ding J. (2014). Experiment research on the relationship of availability and fraction transformation of copper and zinc with pH. Sci. Technol. Eng..

[42] Liu Q.Q., Meng F.P., Lin Y.C. (2019). Bioavailability and ecological risk assessment of heavy metals in sediments of Jiaozhou Bay. Periodical Ocean Univ. China.

[43] Tong M., Wang H., Zhang W.S., Wang C., Song J.X. (2025). Distribution characteristics of bacterial antibiotic resistance genes in heavy metal-contaminated soils. Biodivers. Sci..

[44] Phan K., Ferenci T. (2013). A design-constraint trade-off underpins the diversity in ecologically important traits in species *Escherichia coli*. ISME J..

[45] Chen Z., Zhang W., Wang G., Zhang Y., Gao Y., Boyd S.A., Teppen B.J., Tiedje J.M., Zhu D., Li H. (2017). Bioavailability of Soil-Sorbed Tetracycline to Escherichia coli under Unsaturated Conditions. Environ. Sci. Technol..

[46] Bourguignon T., Kinjo Y., Villa-Martín P., Coleman N.V., Tang Q., Arab D.A., Wang Z., Tokuda G., Hongoh Y., Ohkuma M. (2020). Increased Mutation Rate Is Linked to Genome Reduction in Prokaryotes. Curr. Biol..

[47] Liu W., Wang H., Chen X., Yang D., Kuang G., Sun Z. (2009). Progress on Degradation of Antibiotics in Environment. Prog. Vet. Med..

[48] Wang J., Pan J., Liu F. (2024). Study on the characteristics and mechanism of antibiotic pollution in different aquifers. Hydrogeol. Eng. Geol..

[49] Chen S., Zhou Y., Chen Y., Gu J. (2018). fastp: an ultra-fast all-in-one FASTQ preprocessor. Bioinformatics.

[50] Li H., Durbin R. (2009). Fast and accurate short read alignment with Burrows-Wheeler transform. Bioinformatics.

[51] Li D., Liu C.M., Luo R., Sadakane K., Lam T.-W. (2015). MEGAHIT: an ultra-fast single-node solution for large and complex metagenomics assembly via succinct de Bruijn graph. Bioinformatics.

[52] Fu L., Niu B., Zhu Z., Wu S., Li W. (2012). CD-HIT: accelerated for clustering the next-generation sequencing data. Bioinformatics.

[53] Kang D.D., Froula J., Egan R., Wang Z. (2015). MetaBAT, an efficient tool for accurately reconstructing single genomes from complex microbial communities. PeerJ.

[54] Parks D.H., Imelfort M., Skennerton C.T., Hugenholtz P., Tyson G.W. (2015). CheckM: assessing the quality of microbial genomes recovered from isolates, single cells, and metagenomes. Genome Res..

[55] Parks D.H., Chuvochina M., Rinke C., Mussig A.J., Chaumeil P.A., Hugenholtz P. (2022). GTDB: an ongoing census of bacterial and archaeal diversity through a phylogenetically consistent, rank normalized and complete genome-based taxonomy. Nucleic Acids Res..

[56] Bankevich A., Nurk S., Antipov D., Gurevich A.A., Dvorkin M., Kulikov A.S., Lesin V.M., Nikolenko S.I., Pham S., Prjibelski A.D. (2012). SPAdes: a new genome assembly algorithm and its applications to single-cell sequencing. J. Comput. Biol..

[57] Li Y., Hu X., Dong X. (2020). Preliminary analysis of ecological landscape pattern changes in Baihe Nature Reserve. J. Sichuan For. Sci. Technol..

[58] Minkin I., Medvedev P. (2020). Scalable multiple whole-genome alignment and locally collinear block construction with SibeliaZ. Nat. Commun..

[59] Ministry of Agriculture and Rural Affairs of China (2008).

[60] Ministry of Ecology and Environment of the People's Republic of China (1997).

[61] Ministry of Ecology and Environment of the People's Republic of China (2009).

