# Supplementary material for: Research on the spillover risk of antibiotic resistance gene from the gut microbiome of wild animal: A case study of the golden snub-nosed monkey
Source: iScience. 2026 Sep 24;29(10):117624. doi: 10.1016/j.isci.2026.117624 (PMC13634989; doi:10.1016/j.isci.2026.117624)
Supplement: Supplementary file 1 [file mmc1.pdf]

iScience, Volume *29*

Supplemental information

Research on the spillover risk of antibiotic resistance gene from the gut microbiome of wild animal: A case study of the golden snub-nosed monkey

Sumei Liang, Jiaying Ma, Han Zhang, Hong Li, Shuzhen Zou, and Dayong Li

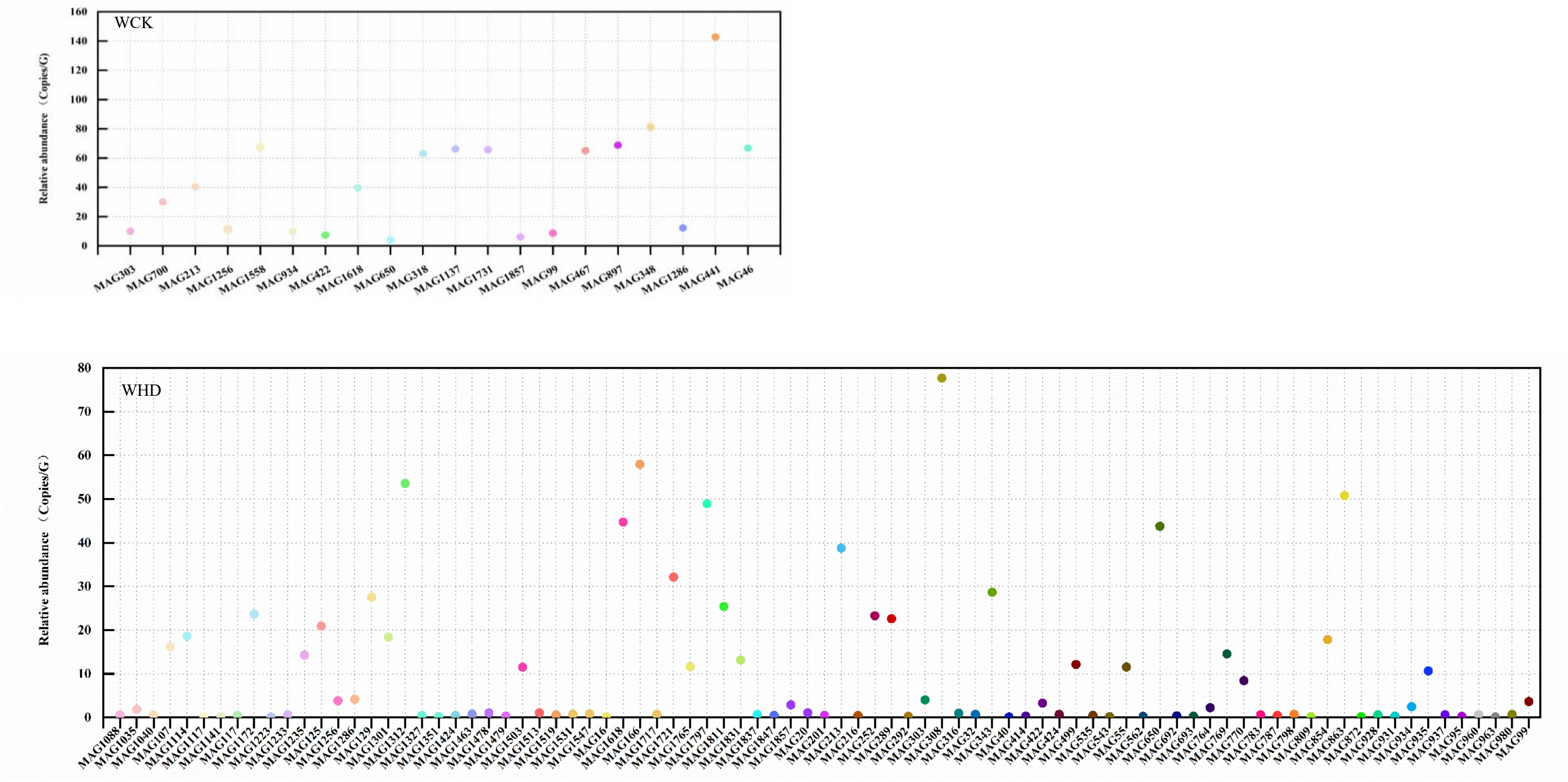

Figure S1. The relative abundance of each MAG in riverine microorganisms

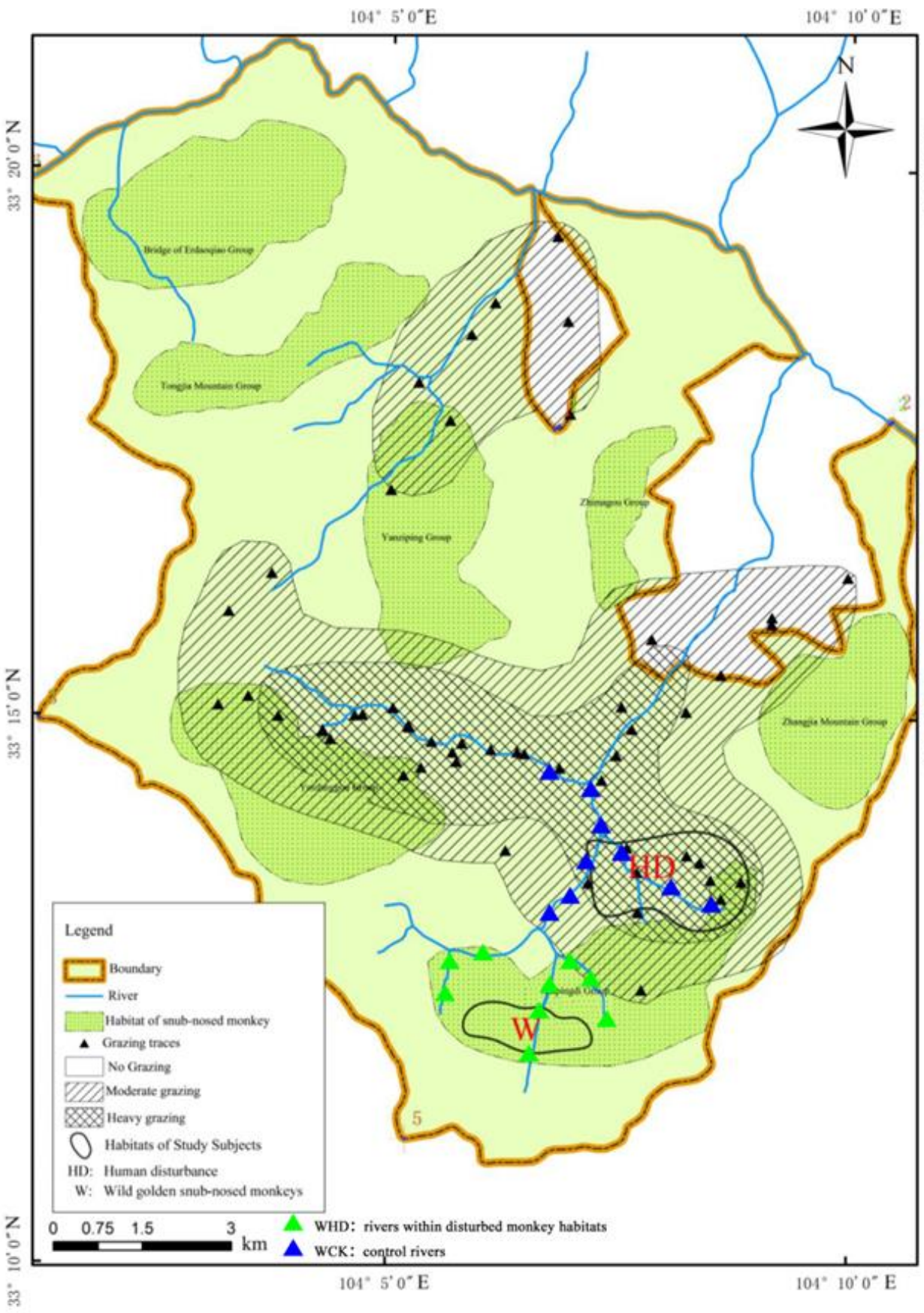

Figure S2. Baihe National Nature Reserve and sampling points.

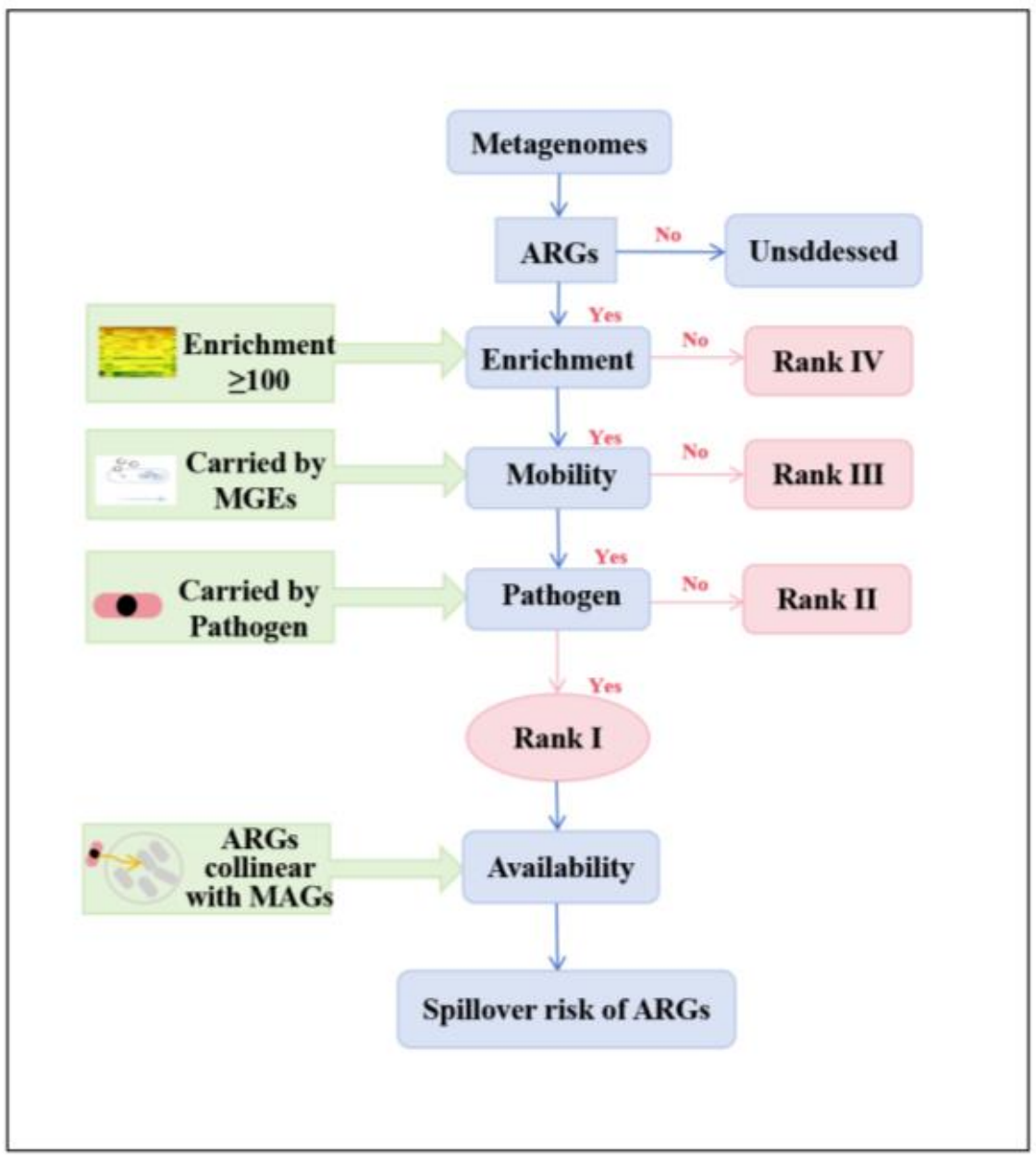

Figure S3. The framework for assessing the spillover risk of ARGs from wild animal gut microbiome to habitat water under human disturbance

Table S1 Statistics of sequences after quality control

Samples	Clean reads	Clean base(bp)	Percent in raw reads(%)	Percent in raw bases(%)
HD1	77476438	11665121980	97.31433742	97.03301402
HD16	83832756	12611947749	98.60231839	98.23779323
HD17	73071860	10994217984	98.54204372	98.1880871
HD31	78923022	11872713675	98.58851191	98.2190326
HD33	85764264	12919630842	98.69521198	98.46069034
HD34	80418470	12084011515	98.16204971	97.68367617
HD35	73325828	11044487264	98.60785702	98.36105004
HD38	80956342	12186912768	98.40843661	98.10659688
HD4	72992046	10975327223	97.89285337	97.48010318
HD40	88227076	13205347635	98.53570619	97.67077608
HD41	80315616	12038928911	98.16433431	97.44613847
HD44	76868064	11559141401	98.22050677	97.81486427
HD47	72785714	10925360293	98.32293	97.73890876
HD50	79125820	11911532795	97.92879981	97.62991511
HD7	86666450	13030676491	97.47657705	97.05977457
W10	86080508	12937342630	97.35532341	96.89983004
W13	100906092	15137673515	98.95139816	98.30751888
W15	79963910	12031380524	98.58435547	98.2318889
W21	76762992	11562064535	97.78286131	97.53697657
W3	92980166	13938718496	98.99361217	98.27945833
W34	96919688	14571371753	98.16837202	97.74241663
W36	77304180	11640967423	98.33832483	98.06904693
W38	83656112	12496201163	99.00686739	97.94194033
W40	98464204	14747709438	98.85876515	98.05831635
W41	86992528	13058778843	98.18889143	97.61263238
W42	80928194	12152694779	98.36261306	97.8195931
W44	78893548	11860575592	98.43198131	97.99943185
W5	85657464	12790800124	99.03663613	97.93804566
W7	77576672	11650926130	97.44229506	96.9169774
W9	79465778	11962084984	97.74670978	97.44329123
WCK1	79411322	11969099292	98.49583818	98.31504374
WCK2	81074228	12220114913	98.37822364	98.20068044
WCK3	74189902	11182470405	98.28507356	98.10780966
WCK4	86864648	13094951600	99.18360255	99.02019217
WCK5	78655928	11856036370	99.2331477	99.05761875
WCK6	89784942	13534980503	99.14364176	98.978769
WCK7	80725896	12169541772	99.23860342	99.07522065
WCK8	87309564	13162768189	99.18548892	99.02768001
WCK9	86012678	12963967194	99.20034624	99.01744012
WHD1	94100612	14177726147	98.16837777	97.95098383
WHD2	79300256	11949276889	98.62359711	98.41718223
WHD3	71364366	10745974922	97.17067366	96.89975459
WHD4	70003288	10540663185	97.59829408	97.32284066
WHD5	78479474	11817649811	97.92192266	97.65129741
WHD6	77375214	11650935961	98.52723614	98.2512999
WHD7	75766092	11416513019	98.67702925	98.46858751
WHD8	74267930	11187236612	97.83676063	97.59928176
WHD9	72969316	10994595253	98.29468373	98.08261887
